# The Necessity of Preparatory Treatment for Child-Bed

**Published:** 1886-09

**Authors:** 


### *THE NECESSITY OF PREPARATORY TREATMENT FOR CHILD-BED.*

In the August number of the *American Journal of Obstetrics*, Dr. H. M Cutts, of Washington, D. C., gives his views upon this important subject. He believes that the pregnant woman should be under the direct supervision of her medical attendant during the last month of pregnancy, at least. Many abnormal conditions may exist, and frequently do exist, which can be corrected to the benefit of mother, child and accoucheur, when labor comes. He should know of her general health, of inherited and acquired diseases, of the diseases and physiological perturbations of pregnancy, the shape and capacity of the pelvis, the abdomen and vagina, and the position, condition and number of fœtuses.

He believes that the profession should educate women up to the idea of the necessity of early engaging the physician who is to attend them, and be under his close direction. He is aware that the system prevails in many communities, but he does not think that the profession carry their investigations far enough. He insists upon the necessity of making a close examination of the genitalia and pelvis, to ascertain all the facts concerning the individual case possible.

"Undoubtedly there are objections. It entails extra labor on the attendant, and expense on the patient. The first item will assuredly not count against it. The second, together with ignorance and negligence on the mother's part, will be its chief opponents. But make it the custom and ignorance cannot be pleaded. Educate womankind up to believe it is for her good, and expense will not stand in the way."

We think that the author could well have added the necessity of giving some special attention to the size, shape and condition of the nipples of primipara, especially. They are too frequently neglected till the time comes for them to be put in use, when they are found sadly wanting. Too much of wheal or woe, for both mother and child, hinge upon the beast and nipples, to trust them exclusively to Dame Nature. Many hours of tedious labor, getting the new comer to nurse, many hours of pain and torture for the mother, and many cases of mastitis may thus be prevented.

